# Dual Circular Polarized Drone-Borne SAR for Polarimetric Target Classification: System Development and Experimental Validation

**DOI:** 10.3390/s26134248

**Published:** 2026-07-04

**Authors:** Dimas Biwas Putra, Yuta Izumi, Fathin Nurzaman, Josaphat Tetuko Sri Sumantyo, Joko Widodo, Shima Kawamura

**Affiliations:** 1 Graduate School of Engineering, Muroran Institute of Technology, Muroran 050-8585, Japan; 24096510@muroran-it.ac.jp (D.B.P.); 23096514@muroran-it.ac.jp (F.N.); skawamur@muroran-it.ac.jp (S.K.); 2 Department of Electrical Engineering, Universitas Sebelas Maret, Surakarta 57126, Indonesia; jtetukoss@faculty.chiba-u.jp; 3 Center for Environmental Remote Sensing, Chiba University, Chiba 263-8522, Japan; 4 National Research and Innovation Agency (BRIN), Jakarta 10340, Indonesia; joko.widodo@brin.go.id

**Keywords:** drone-borne SAR, post-disaster assessment, motion compensation, TDBP, PGA, dual circularly polarized mode (DCP), *H*/*α* decomposition, polarimetric calibration

## Abstract

Post-disaster scenarios such as tsunamis require rapid terrain assessment that cannot wait for the next satellite synthetic aperture radar (SAR) revisit, yet a readily deployable system remains lacking. We present an off-the-shelf K-band drone-borne dual circular polarimetric (DCP) SAR and a processing pipeline for on-demand terrain classification. Compared with fully polarimetric (FP) SAR, DCP requires only a single transmit polarization and two receive channels, providing a wider swath than FP for the same acquisition, while still separating odd-bounce and even-bounce scattering mechanisms, which dual linear polarimetric modes with the same channel count provide with greater ambiguity due to their sensitivity to target orientation angle. To compensate for platform motion, we implemented RTK global navigation satellite system (GNSS) guided time-domain backprojection (TDBP) with phase gradient autofocus (PGA), yielding an 11.98 dB improvement in peak amplitude. We then applied single-target wire calibration to correct a measured 8.91 dB inter-channel complex gain difference between co-polarization and cross-polarization. As a result, *H*/*α* decomposition of the calibrated DCP data classifies canonical reflectors, artificial structures, gravel roads, vegetation, and a pond surface. These field experiments extend compact polarimetric *H*/*α* decomposition to drone-borne SAR data for terrain discrimination, establishing a practical pathway toward rapid post-disaster terrain assessment.

## 1. Introduction

Synthetic aperture radar (SAR) is a well-established remote sensing technology that enables all-weather, day-and-night imaging for terrain mapping, environmental monitoring, and surveillance [1,2]. Conventional SAR systems on satellites or aircraft provide wide-area coverage but are constrained by revisiting time, flexibility, and cost. In recent years, drone-borne SAR has emerged as an attractive alternative. Svedin et al. [3] demonstrated a small drone-borne SAR using a low-cost 5–6 GHz radar with global navigation satellite system (GNSS) RTK positioning and onboard image generation. Bekar et al. [4] demonstrated very fine cross-range imaging using a 77 GHz automotive radar mounted on a hexacopter in controlled experiments. Lort et al. [5] evaluated polarimetric and interferometric modes on a multicopter at 10 GHz. While these works established drone-borne SAR systems and demonstrated polarimetric data acquisition, motion compensation, and system validation, they did not extend the acquired polarimetric data to *H*/*α* target decomposition for terrain classification, which is the focus of this work. The availability of commercial radar modules, low-cost GNSS RTK systems, and lightweight computers continues to accelerate platform development [6]. However, small drones are susceptible to wind, turbulence, and vibrations that degrade image quality through phase errors and range cell migration [4,7]. To address this, various motion compensation and autofocus strategies have been developed, including improved phase-correction methods, geometrical and sharpness-based autofocus techniques, time-domain back-projection (TDBP), and GNSS-based positioning strategies [7,8,9,10,11,12,13,14,15].

These developments are enabling drone-borne SAR to be deployed in operationally demanding scenarios, among which post-disaster environmental monitoring is particularly compelling. Polarimetric SAR has proven effective for this purpose: fully polarimetric (FP) SAR measures the complete scattering matrix, enabling target decomposition techniques such as the *H*/*α* decomposition and model-based three-component and four-component scattering power decompositions to classify targets based on their scattering mechanisms [16,17]. Park et al. [18] evaluated polarimetric change detection using spaceborne L-band ALOS/PALSAR data for automatic identification of damage caused by the 2011 Tohoku earthquake and tsunami, demonstrating that polarimetric parameters achieved detection accuracy of approximately 90%, while Chen and Sato [19] demonstrated that multitemporal FP SAR can quantify tsunami damage in built-up areas using scattering mechanism changes. While these studies highlight the power of FP SAR for damage assessment, rapid situational awareness remains critical for response and recovery [20]. Satellite SAR may delay acquisition by hours or days due to fixed revisit intervals [1], whereas drone-borne SAR enables on-demand imaging within minutes of deployment. Its low-altitude operation provides a higher signal-to-noise ratio, and the platform can be redeployed repeatedly to monitor evolving conditions such as flood extent, structural damage, or terrain changes [20,21]. Garcia-Fernandez et al. [21] and Colorado et al. [22] demonstrated that drone-borne SAR can also serve safety-critical field applications such as landmine detection using ground-penetrating radar. Luebeck et al. [23] further extended capabilities by demonstrating drone-borne DInSAR for surface displacement measurement. These studies confirm that drone-borne SAR is well suited for rapid terrain assessment.

To fully exploit drone-borne SAR for terrain classification, polarimetric information is essential. This capability is widely used for land-cover classification, vegetation structure analysis, and post-disaster damage assessment. However, FP SAR has been rarely used for emergency operations after disasters, primarily due to operational constraints such as narrower swath width compared to single- or dual-polarization modes [1].

**Figure 1:**
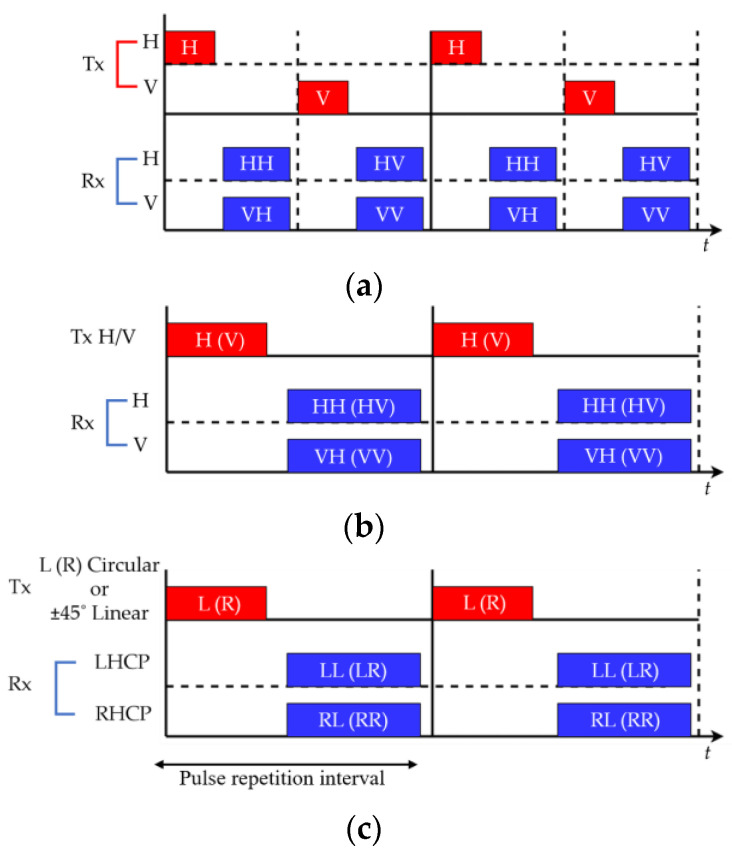
Tx and Rx timing for three polarimetric modes, all with dual-channel reception: (**a**) FP, alternating H/V transmit (four channels); (**b**) DLP, single linear transmit; (**c**) CP, single circular or ±45° linear transmit (LL/RL channels). Modified after [1].

Compact polarimetric (CP) SAR addresses these limitations by transmitting a single polarization and receiving two orthogonal channels simultaneously. As illustrated in Figure 1, FP (Figure 1a), DLP (Figure 1b), and CP (Figure 1c) modes differ in their Tx/Rx timing. Because FP must transmit two polarizations in alternation, whereas CP transmits only one, CP achieves a wider swath than FP for the same acquisition [24,25], using a single transmit chain. Moreover, CP modes can distinguish odd-bounce and even-bounce scattering, a capability that dual linear polarimetric (DLP) modes (Figure 1b) with the same number of channels can also provide but with greater ambiguity, as DLP-based separation through amplitude ratios and phase analysis remains sensitive to target orientation angle, which can cause misclassification when targets are not aligned with the polarization basis [1,26,27]. Several CP architectures have been discussed: Souyris et al. [25] introduced the π/4 mode, Stacy et al. [28] discussed the dual circularly polarized (DCP) mode, and Raney [24] introduced the hybrid-polarity (CTLR) architecture. Although these modes differ in polarization basis, both circular and ±45° rotated linear transmit polarizations produce a coherent combination of H and V components, making them theoretically equivalent for CP decomposition; Nord et al. [26] further showed that they provide similar scattering retrieval performance in practice. In this work, we adopt the DCP mode for the drone-borne SAR system.

Several studies have adapted the *H*/*α* decomposition for CP data to improve scattering characterization. Raney et al. [29] proposed the *m–χ* decomposition as an alternative interpretative framework, Zhang et al. [30] adapted *H*/*α* for CP and demonstrated its effectiveness in distinguishing scattering mechanisms; and Buono et al. [31] applied CP analysis to detect sea oil slicks. For canonical targets measured in the DCP mode, the alpha angle follows the complementary relation $\alpha_{DCP}=90^{\circ }-\alpha_{FP}$ [1,32], which provides a theoretical basis for comparing measurement modes. Despite these advances, no study to date has applied compact polarimetric *H*/*α* target decomposition to data acquired from drone-borne SAR platforms.

In this paper, we address this gap by presenting a processing pipeline for an off-the-shelf K-band drone-borne SAR that enables polarimetric terrain classification. A practical single-target wire calibration is applied to correct inter-channel complex gain differences (amplitude and phase) between the co- and cross-polarized channels before *H*/*α* decomposition for scattering-mechanism identification. The system transmits a single LHCP (left-hand circular polarization) signal and simultaneously receives LHCP and RHCP using circularly polarized antennas mounted on a drone. Field measurements show that canonical reflectors yield alpha angles consistent with theoretical predictions, and that terrain classes, artificial structures, gravel roads, vegetation, and a pond surface occupy distinct regions in the *H*/*α* plane. To the best of the authors’ knowledge, this is the first application of compact polarimetric *H*/*α* target decomposition to drone-borne SAR data for on-demand terrain classification.

By combining polarimetric classification capability with the rapid-deployment capabilities of a drone platform, this work offers a practical pathway toward faster and more informative post-disaster terrain assessment than current single-polarization drone SAR or revisit-constrained satellite systems can provide.

## 2. System Development

**Figure 2:**
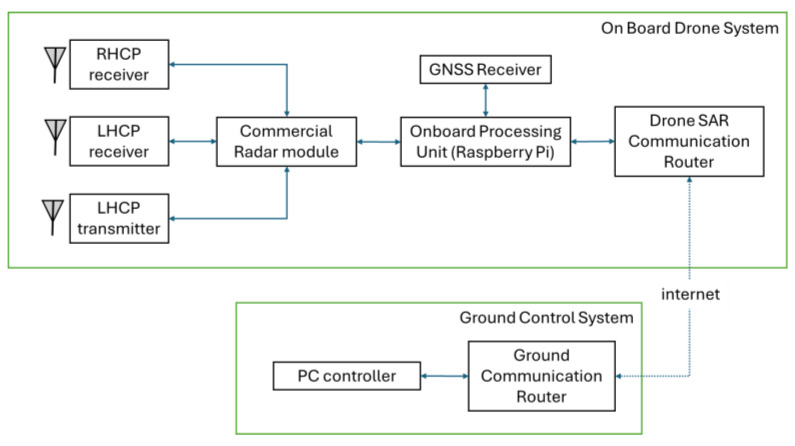
K-Band drone-borne SAR hardware block diagram.

The proposed drone-borne SAR system integrates a K-band frequency-modulated continuous-wave (FMCW) radar with circular polarization onto a drone platform. Figure 2 illustrates the complete system configuration deployed for the experiments.

**Figure 3:**
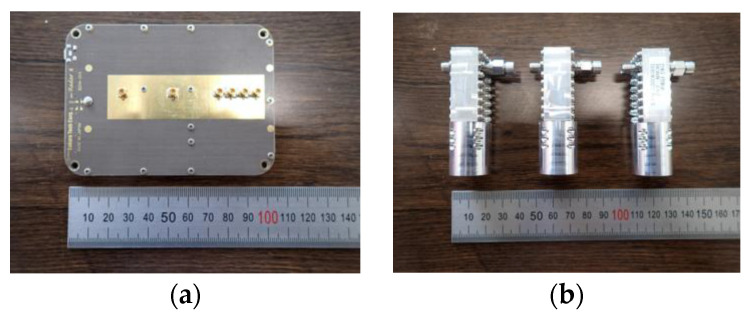
(**a**) K-band commercial radar module and (**b**) circularly polarized horn antenna.

The drone platform is a DJI Matrice 300, selected for its payload capacity and flight stability [33]. The K-band commercial radar module (Figure 3a) operates at a center frequency of 24.15 GHz with a bandwidth of 180 MHz, providing a theoretical range resolution of approximately 0.83 m. The module features interchangeable antenna ports, to which circularly polarized horn antennas (Figure 3b) are connected. Each horn antenna can be configured for either LHCP or RHCP. The system employs a single LHCP transmit antenna and dual receive antennas configured for both RHCP and LHCP reception, enabling DCP measurements: RL (RHCP-receive, LHCP-transmit, cross-polarization) and LL (LHCP-receive, LHCP-transmit, co-polarization). The radar’s sampling rate is set to 1.8 MHz.

**Figure 4:**
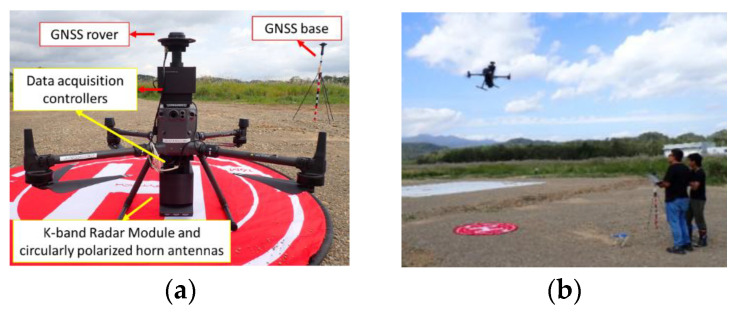
DCP drone-borne SAR system. (**a**) SAR unit with rover and base-station GNSS receiver. (**b**) Flight experiment during drone operation.

The radar unit is mounted beneath the drone using a custom holder angled at 45 degrees relative to the flight direction to achieve the desired imaging geometry. A Raspberry Pi single-board computer serves as the data acquisition controller, managing radar timing and data storage during flight operations. All SAR processing is performed post-flight on a ground station; the Raspberry Pi onboard serves solely as data acquisition controller. Precise positioning is achieved through a GNSS rover module operating in real-time kinematic (RTK) mode, providing horizontal positional accuracy of approximately ±1 cm and vertical accuracy of 3–5 cm. The RTK solution is updated at a maximum frequency of 5 Hz (once every 0.2 s). All system components are powered by a dedicated onboard battery, separate from the drone’s propulsion battery, to ensure stable power supply during data acquisition. A photograph of the complete drone-borne SAR unit is shown in Figure 4a, while Figure 4b presents an example of the flight experiment conducted in the open field. The specifications of all hardware components are summarized in Table 1.

While the hardware configuration described above provides the necessary measurement capability, the use of an off-the-shelf commercial FMCW radar introduces hardware-induced amplitude and phase instabilities arising from power-supply fluctuations, oscillator drift, and internal timing imperfections [34,35]. These imperfections must be corrected before SAR processing to ensure coherent image formation. In our system, the acquired radar data revealed a periodic error pattern affecting both amplitude and phase, a known characteristic of commercial FMCW radar modules arising from internal timing cycles [34,35,36]. The amplitude and phase corrections were applied to produce the final calibrated complex beat signal.

## 3. Methodology and Simulation

**Figure 5:**
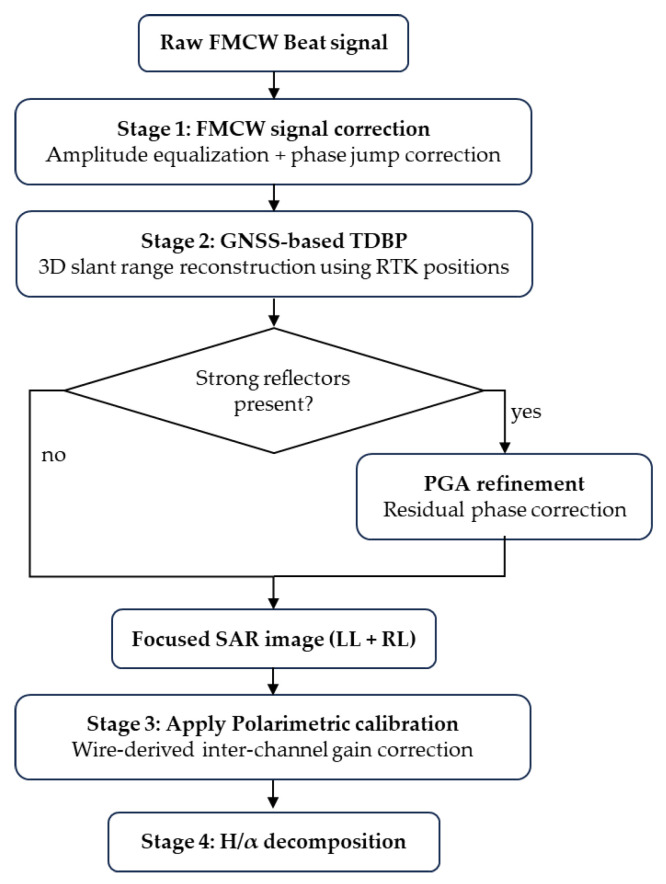
Processing pipeline of the DCP drone-borne SAR system.

The overall processing pipeline for the DCP drone-borne SAR system is illustrated in Figure 5 and consists of four stages. First, the raw radar beat signal undergoes amplitude and phase corrections to produce a calibrated complex beat signal. Second, motion compensation is performed using GNSS-based TDBP, optionally followed by phase gradient autofocus (PGA) for scenes containing prominent scattering. Third, the polarimetric calibration factor derived from a wire reflector measurement is applied to correct inter-channel complex gain differences between co- and cross-polarized channels. Fourth, *H*/*α* decomposition is applied to the calibrated DCP data to extract scattering mechanism parameters for terrain classification.

### 3.1. Motion Compensation in Imaging

In an FMCW radar, the transmitter emits a frequency-swept chirp signal, and the receiver mixes the returned echo with a copy of the transmitted waveform to produce a beat signal whose frequency is proportional to the target range. Before SAR focusing is applied, this raw beat signal must be converted to a range-compressed profile (beat spectrum) by discrete Fourier transform. The collection of all pulses along the flight track forms the two-dimensional beat spectrum dataset $S\left(x_{q},r_{q}\right),$ with $x_{q}$ and $r_{q}$ representing cross-range position and the slant range distance, where q indicates transmit index, used for image formation. The image grid pixel spacing is set to *λ*/2 tan(*θ*) in cross-range and dr/2 in range, where *θ* is the antenna half-beamwidth, and dr is the range resolution, with image dimensions varying per scene depending on the flight track length and scene extent.

For image reconstruction, this study applies the TDBP algorithm to the beat spectrum dataset. TDBP reconstructs the complex reflectivity at each grid point by coherently summing the received radar signals from all antenna positions along the synthetic aperture. The reconstructed reflectivity ${\hat{\sigma }}_{TDBP}$ at arbitrary grid point ($x_{i},y_{i}$) is given by

(1) $${\hat{\sigma }}_{TDBP}\left(x_{i},y_{i}\right)=\sum_{q=1}^{Q}S\left(x_{q},r_{q}^{\prime }\right)\exp\left(-j\frac{4\pi r_{q}^{\prime }}{\lambda }\right)$$

where $r_{q}^{\prime }=\sqrt{{\left(x_{q}-x_{i}\right)}^{2}+y_{i}^{2}+z_{i}^{2}}$ is the slant range from the antenna position at $x_{q}$ to the grid point ($x_{i},y_{i},z_{i}$); λ is the radar wavelength at the center frequency; and Q is the total number of transmissions in the synthetic aperture.

**Figure 6:**
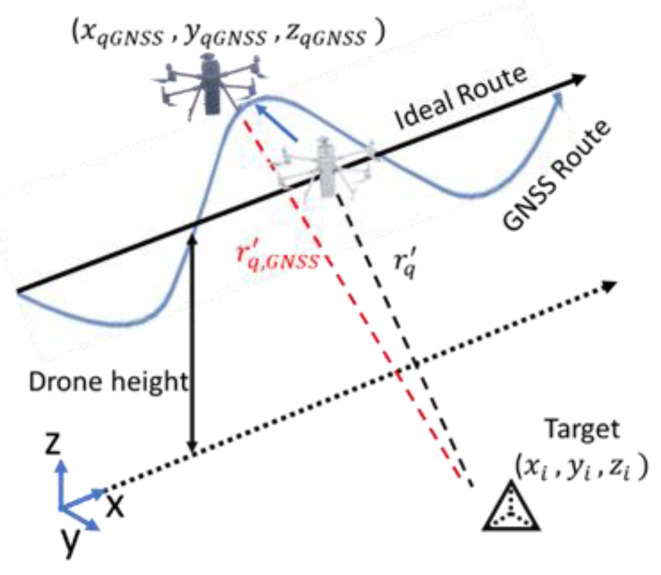
Imaging geometry in the three-dimensional coordinate system. The black and red dashed lines denote $r_{q}^{\prime }$ and $r_{q,GNSS}^{\prime }$, respectively.

TDBP allows straightforward incorporation of GNSS data for motion compensation. By utilizing the recorded three-dimensional antenna positions from the RTK GNSS receiver, the slant range can be computed directly for each grid point, accounting for the full spatial trajectory of the drone [37]. Figure 6 illustrates the imaging geometry and the GNSS-corrected slant range computation, where x, y, and z denote the cross-range, range, and altitude axes, respectively. With the GNSS-recorded positions, the slant range $r_{q,GNSS}^{\prime }$ can be redefined as

(2) $$r_{q,GNSS}^{\prime }=\sqrt{{\left(x_{q,GNSS}-x_{i}\right)}^{2}+{\left(y_{q,GNSS}-y_{i}\right)}^{2}+{\left(z_{q,GNSS}-z_{i}\right)}^{2}}$$

where ${(x}_{q,GNSS},y_{q,GNSS},z_{q,GNSS})\;$are the three-dimensional coordinates of the SAR platform at index q, obtained from GNSS measurements. The 45° antenna tilt angle is incorporated directly into the TDBP geometry through the three-dimensional slant range computation in Equation (2), which accounts for the full antenna position vector. By incorporating all three spatial components of the drone position into the slant range computation, the GNSS-based TDBP inherently compensates for cross-track drift and altitude variations during flight [37]. The GNSS coordinates, recorded at a 5 Hz update rate, are linearly interpolated in three-dimensional Cartesian coordinates to match the approximately 400 Hz transmission repetition frequency.

While the manufacturer half-power beamwidth is 45°, an effective 40° is used in the TDBP image formation, beyond which the coherent contribution is insufficient for focusing; the imaged beam thus spans incidence angles from 25° to 65°. The resulting ground-range ($W_{g})$ and slant-range swaths ($W_{s}$) scale with platform altitude $Z_{0}$ as $W_{g}$ = $Z_{0}$(tan 65°−tan 25°) = 1.68  $Z_{0}$, $W_{s}$ = $Z_{0}$ (sec 65°−sec 25°) = 1.26  $Z_{0}$. For the altitudes used, $Z_{0}$ = 10 m (natural-scene and canonical-target experiments) and $Z_{0}$ = 15 m (artificial-structure), the ground-range swaths are 16.8 m and 25.2 m, and the slant-range swaths are 12.6 m and 18.9 m, spanning slant ranges of 11.0–23.7 m and 16.6–35.5 m, respectively.

While the GNSS-based TDBP algorithm compensates for the geometric motion errors captured by the RTK GNSS, residual phase errors may persist due to several factors: (1) GNSS measurement noise at the centimeter level, (2) interpolation errors when matching the 5 Hz GNSS rate to the ~400 Hz pulse repetition frequency (PRF), and (3) high-frequency platform vibrations not captured by the GNSS sampling rate. These residual errors can degrade the image focus, particularly for high-resolution imaging applications. To mitigate these residual errors, data-driven autofocus algorithms are employed.

**Figure 7:**
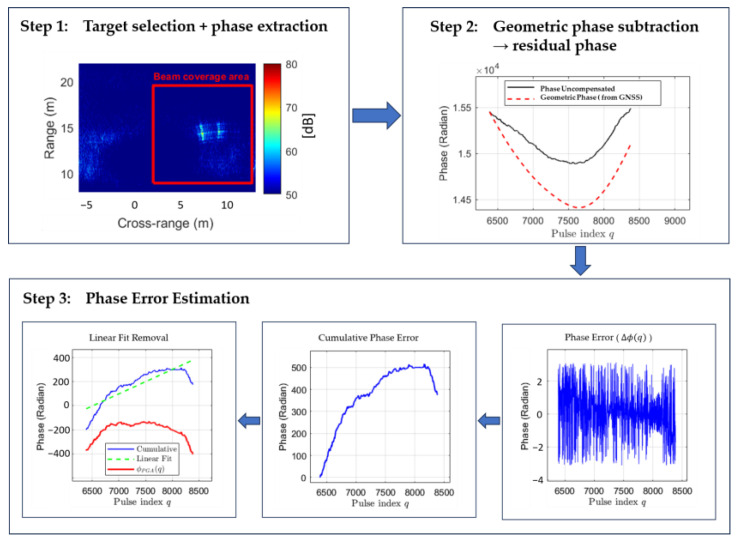
Step-by-step procedure of PGA.

In this work, a PGA algorithm [10] is applied after the initial TDBP image formation to correct residual phase errors that remain after GNSS-based motion compensation. The procedure implemented in this study consists of three steps, as illustrated in Figure 7.

In step 1, a prominent point-like scatterer is selected from the initial TDBP image. For each radar pulse whose antenna beam covers the selected target position (indicated by the red box in Figure 7, step 1), the beat spectrum is read at the slant range corresponding to the target.

In step 2, the phase of the extracted signal and the geometric phase computed from the GNSS trajectory are compared, as shown in Figure 7 (step 2). The geometric phase represents the expected two-way propagation phase between the antenna and the target, as expressed in Equation (1). If the GNSS positions were perfectly accurate, both phases would be identical across all pulses. The difference between these two phases constitutes the residual phase, whose fluctuations directly reflect the position errors in the GNSS-measured trajectory.

In step 3, a continuous phase error function is estimated from the residual phase following the phase gradient approach [10], through three sub-steps as illustrated in Figure 7 (step 3). First, the inter-pulse phase error is computed between consecutive pulses:

(3) $$\Delta \phi \left(q\right)\;=\;(\Psi_{uncomp}\left(q\right)\;\;-\;\Psi_{uncomp}\left(q-1\right))\;-\;(\;\Psi_{GNSS}\left(q\right)\;-\;\Psi_{GNSS}\left(q-1\right))$$

where $\Psi_{uncomp}\left(q\right)$ is the measured phase uncompensated, and $\Psi_{GNSS}\left(q\right)$ is the geometric phase from the GNSS trajectory at pulse *q*. When multiple reference targets are selected, the Δϕ values from each target are combined through weighted averaging, following the principle in [10] where the redundancy of the common phase error across multiple scatterers is exploited to improve the estimation accuracy. Second, the Δϕ values are integrated via cumulative summation to reconstruct the cumulative phase error along the synthetic aperture; the effect of optionally smoothing the gradients prior to integration is examined in the Section 4. Third, a first-order polynomial fit is subtracted from the cumulative phase error to remove the linear trend and mean offset, eliminating the low-frequency geometric component that would otherwise cause a spatial shift of targets in the final image. The resulting zero-mean function, $\phi_{PGA}\left(q\right)$, represents the estimated phase error used for the subsequent compensation.

At this stage, the estimated phase error function is ready to be incorporated into the image formation process, where it serves as the per-pulse correction term required to restore coherent summation across the aperture. The estimated phase correction is applied on a per-pulse basis during a final TDBP image formation. Each pulse’s contribution is multiplied by the conjugate of the estimated phase error, compensating for residual phase variations so that signals from the target add coherently, resulting in a sharper, better-focused image with improved peak response. The resulting correction $\phi_{PGA}\left(q\right)$ is applied directly within the back-projection loop on a per-pulse basis:

(4) $${\hat{\sigma }}_{corrected}\left(x_{i},y_{i}\right)=\sum_{q=1}^{Q}S\left(x_{q},r_{q}^{\prime }\right)\exp\left(-j\frac{4\pi r_{q}^{\prime }}{\lambda }\right)\cdot \exp\left(-j\phi_{PGA}\left(q\right)\right)$$

where $\phi_{PGA}\left(q\right)$ is the estimated residual phase error at pulse *q*, obtained through the procedure described above.

**Figure 8:**
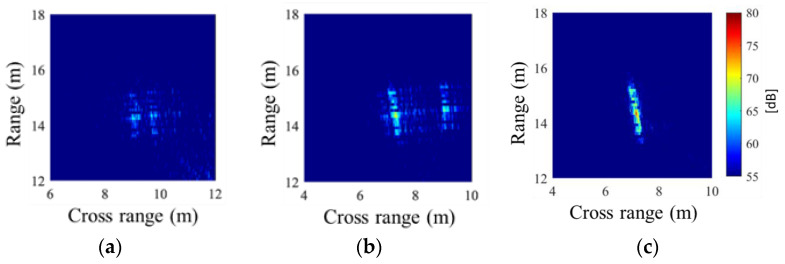
Comparison of focusing results. (**a**) No motion compensation. (**b**) GNSS-based motion correction. (**c**) GNSS plus PGA correction.

**Figure 9:**
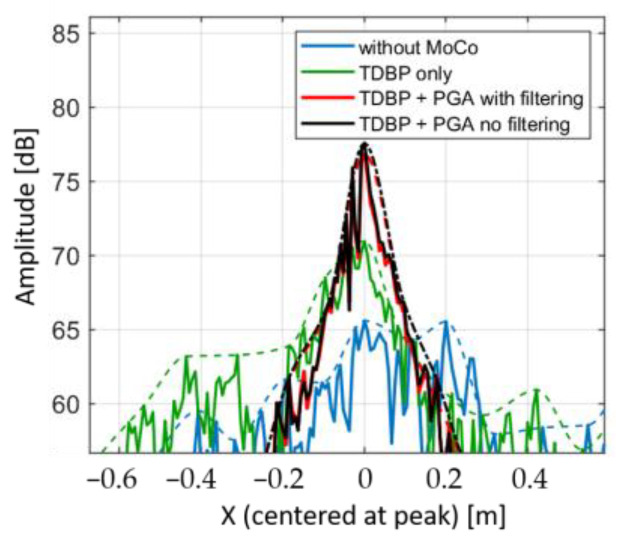
Power profile comparison at a dihedral target: without motion compensation (blue), with GNSS-based 3-axis TDBP (green), with GNSS-based 3-axis TDBP + PGA filtered (red), and with GNSS-based 3-axis TDBP + PGA unfiltered (black).

To illustrate the effect of motion compensation, we evaluated the method on a drone-borne SAR dataset containing both dihedral and trihedral corner reflectors, which are described in detail in Section 4. All quantitative results reported below are derived from the LL channel, which isolates the dihedral response used for the comparative analysis. In contrast, the trihedral response appears predominantly in the RL channel. Figure 8 shows the LL-channel image used for this comparison, where Figure 8a presents the result without motion compensation, Figure 8b after GNSS-based TDBP correction, and Figure 8c after GNSS plus PGA correction. The horizontal axis corresponds to cross-range, and the vertical axis to range; the image displays the region surrounding the deployed targets. Without motion compensation, the LL image exhibits a peak amplitude of 65.58 dB and a half power beam width (HPBW) of 8.75 cm, indicating limited coherent integration due to uncompensated platform motion. Applying GNSS-based 3-axis TDBP increases the peak amplitude to 70.95 dB and simultaneously narrows the HPBW to 7.98 cm, demonstrating that the three-dimensional trajectory correction improves imaging quality. It should be noted that PGA requires the presence of prominent point-like targets in the scene for reliable phase error estimation. For environmental scenes that lack such dominant scatterers, GNSS-based TDBP alone provides motion correction without the PGA refinement step, as demonstrated by the green curve in Figure 9, which shows meaningful improvement over the uncompensated case.

The subsequent application of PGA further enhances the image, raising the peak amplitude to 77.56 dB and sharply reducing the HPBW to 2.32 cm (Figure 9). These results confirm that GNSS-guided TDBP provides substantial geometric correction, while PGA effectively compensates higher-order residual phase errors that remain after trajectory correction. Unlike standard PGA, which typically applies filtering to the phase gradient, both filtered and unfiltered corrections were evaluated here, yielding comparable results (peak difference < 1 dB, HPBW difference < 0.1 cm), as shown in Figure 9. In this implementation, the filtering consists of a moving-average smoothing of the phase gradient prior to its integration into the phase-error estimate. The unfiltered version was adopted as it yields a marginally superior peak response; the small difference between the two indicates that the high-frequency content removed by the filter has little effect on the focused image, consistent with the suppression of high-frequency gradient noise by the subsequent integration.

The combined 3-axis TDBP and PGA processing yields a total peak gain of +11.98 dB relative to the uncompensated case and reduces the HPBW from 8.75 cm to 2.32 cm, approaching the theoretical resolution limit imposed by the system bandwidth. These results confirm that integrating geometric motion correction with phase-based refinement is essential for recovering coherent energy and achieving high-resolution SAR imaging on drone-borne platforms.

### 3.2. Polarimetric Decomposition for Dual-Circular Polarimetric SAR

#### 3.2.1. Wave Coherency Matrix and Stokes Parameters

The polarimetric analysis begins with the formulation of the DCP scattering vector and wave coherency matrix, from which the *H*/*α* decomposition parameters are derived. The DCP scattering vector for LHCP transmission is defined as [1,32], where j^2^ = −1 denotes the imaginary unit:

(5) $$k_{DCP}=\left[\begin{array}{c} S_{LL}\\ S_{RL} \end{array}\right]=\frac{1}{2}\left[\begin{array}{c} S_{HH}-S_{VV}+2jS_{HV}\\ j\left(S_{HH}+S_{VV}\right) \end{array}\right]$$

This relationship demonstrates the connection between circular polarization (L-R) and linear polarization basis (H-V). The 2 × 2 wave coherency matrix for DCP data can be expressed in terms of the four Stokes parameters ($g_{0}$, $g_{1}$, $g_{2}$, $g_{3}$) as [26]

(6) $${\left[J\right]}_{DCP}=\left[\begin{array}{cc} \langle S_{LL}S_{LL}^{*}\rangle & \langle S_{LL}S_{RL}^{*}\rangle \\ \langle S_{RL}S_{LL}^{*}\rangle & \langle S_{RL}S_{RL}^{*}\rangle \end{array}\right]=\left[\begin{array}{cc} \langle g_{o}\rangle +\langle g_{3}\rangle & \langle g_{2}\rangle +j\langle g_{1}\rangle \\ \langle g_{2}\rangle -j\langle g_{1}\rangle & \langle g_{o}\rangle -\langle g_{3}\rangle \end{array}\right]$$

#### 3.2.2. H/α Decomposition for DCP Mode

The entropy-alpha (*H*/*α*) decomposition provides a robust framework for scattering mechanism identification from polarimetric SAR data [16,32]. This approach, originally developed for fully polarimetric systems by Cloude and Pottier [16], has been successfully adapted for compact polarimetric configurations, including DCP mode [30]. The *H*/*α* decomposition for DCP is based on the eigenvalue decomposition of the wave coherency matrix:

(7) $$\left[J\right]=\left[\begin{array}{ll} {\vec{u}}_{1} & {\vec{u}}_{2} \end{array}\right]\left[\begin{array}{cc} \lambda_{1} & 0\\ 0 & \lambda_{2} \end{array}\right]{\left[\begin{array}{ll} {\vec{u}}_{1} & {\vec{u}}_{2} \end{array}\right]}^{*T}$$

where $\lambda_{1}\geq \lambda_{2}\geq 0$ are the eigenvalues sorted in descending order, and ${\vec{u}}_{i}$ are the corresponding orthonormal eigenvectors. Each eigenvector can be parameterized as

(8) $${\vec{u}}_{i}=\left[\begin{array}{c} cos\;\alpha_{i}\\ sin\;\alpha_{i}\cdot e^{j\delta_{i}} \end{array}\right]$$

where $\alpha_{i}\in \left[0{}^{\circ},90{}^{\circ}\right]$ is the alpha angle associated with the *i*-th scattering mechanism and $\delta_{i}$ is the phase difference. Following the standard eigenvector-based approach [16,32], the alpha angle for each eigenvector is extracted as

(9) $$\alpha_{i}=\sin^{-1}\left(\left|u_{2,i}\right|\right)=\cos^{-1}\left(\left|u_{1,i}\right|\right)$$

where $u_{1,i}$ and $u_{2,i}$ represent the first and second components of the *i*-th unit eigenvector, corresponding to the $S_{LL}$ and $S_{RL}$ channel contributions, respectively. The two formulations are mathematically equivalent since $|u_{1,i}{|}^{2}+|u_{2,i}{|}^{2}=1$ for unit eigenvectors. The scattering probabilities are defined as

(10) $$P_{i}=\frac{\lambda_{i}}{\lambda_{1}+\lambda_{2}}\left(i=1,2\right)$$

The scattering entropy $H_{DCP}$ quantifies the randomness or diversity of scattering mechanisms:

(11) $$H_{DCP}=-\sum_{i=1}^{2}\;P_{i}\log_{2}\left(P_{i}\right)$$

The entropy ranges from 0 to 1, where H≈0 indicates a single dominant scattering mechanism (deterministic target), while H≈1 indicates multiple random scattering mechanisms with equal probability. The mean alpha angle ${\overset{ˉ}{\alpha }}_{DCP}$ characterizes the average scattering mechanism type:

(12) $${\bar{\alpha }}_{DCP}=\sum_{i=1}^{2}\;P_{i}\alpha_{i}$$

A significant advantage of the DCP configuration is its ability to preserve scattering mechanism information comparable to FP systems. For a single canonical target where one eigenvalue dominates (i.e., $\lambda_{1}\neq 0$ and $\lambda_{2}\approx 0$), the relationship between alpha angles of DCP and FP modes can be expressed as [32]

(13) $${\bar{\alpha }}_{DCP}=90^{\circ }-{\bar{\alpha }}_{FP}$$

This relationship yields the correspondence for canonical scattering mechanisms as shown in Table 2.

This correspondence demonstrates that DCP data can effectively discriminate between the three fundamental scattering mechanisms, validating its use as a practical alternative to fully polarimetric systems when payload constraints limit antenna configurations [27,32].

**Figure 10:**
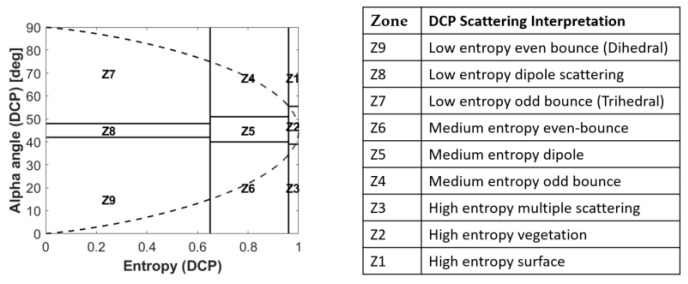
Two-dimensional *H*/*α* classification plane for DCP mode. Modified after [1]. Solid curves indicate the feasible region; dashed lines denote the zone boundaries.

The *H*/*α* plane is divided into nine zones (Z1–Z9) based on entropy and alpha angle boundaries, as shown in Figure 10 [30,32]. Low-entropy zones (Z7, Z8, Z9) correspond to deterministic scattering: Z7 for odd-bounce (trihedral, $\alpha_{DCP}$ ≈ 90°), Z8 for wire ($\alpha_{DCP}$≈ 45°), and Z9 for even-bounce (dihedral, $\alpha_{DCP}\;$≈ 0°). Higher-entropy zones indicate increasingly random or mixed scattering mechanisms.

### 3.3. Polarimetric Calibration Using Wire Reflector

In circular polarization, the wire produces equal magnitude responses in both channels [1,38], as shown by its scattering matrix in the L-R basis:

(14) $$k_{DCP,wire}=\left[\begin{array}{c} S_{LL}\\ S_{RL} \end{array}\right]=\;\frac{1}{2}\left[\begin{array}{c} 1\\ j \end{array}\right]$$

This balanced response makes the wire an ideal calibration reference [39]: any measured deviation from the unity ratio ($|S_{LL}|/|S_{RL}|\neq 1$) directly quantifies the complex gain difference between the two receiver channels.

**Figure 11:**
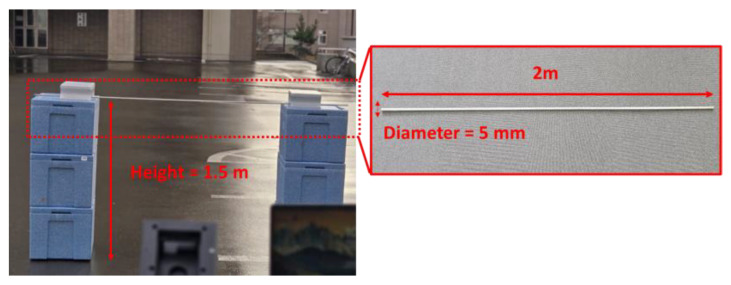
Wire calibration experiment setup with dielectric boxes and wire reflector, where the close-up inset shows the wire detail.

A calibration experiment was conducted using a horizontal wire reflector (length: 2 m, diameter: 5 mm) positioned at 7 m from the radar antenna, as shown in Figure 11. The calibration distance of 7 m is well within the far-field region of the horn antenna used (2D^2^/λ ≈ 0.064 m, where D ≈ 0.02 m is the antenna aperture diameter and λ = 0.0124 m at 24.15 GHz), indicating that near-field effects and antenna pattern variations are negligible at this range [40,41]. The wire was mounted at 1.5 m height on dielectric boxes, with the antenna at the same height to minimize ground reflections within the beamwidth. Measurements were performed with and without the wire present to isolate the wire contribution from background clutter. A wire radius of 0.2*λ* was selected as a compromise: sufficiently thin to make the circular cross-section contribution negligible, yet large enough to maintain adequate signal-to-noise ratio for reliable calibration.

**Figure 12:**
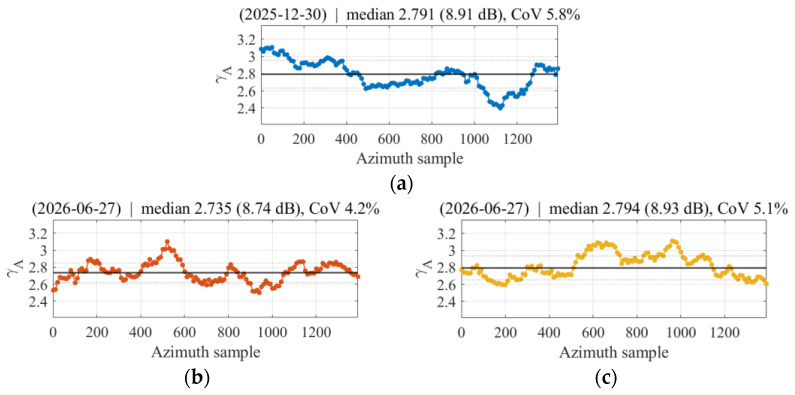
Measured channel imbalance $\gamma_{A}$ over azimuth samples for three calibration sessions: (**a**) 12 December 2025, (**b**) 27 June 2026 (Session A), and (**c**) 27 June 2026 (Session B), confirming hardware response consistency over approximately six months.

From the wire-present and background measurements, the complex calibration factor *γ* is derived:

(15) $$\gamma =\gamma_{A}\;.\;e^{j\left(\gamma_{P}\right)}=\frac{\Delta A_{RL}}{\Delta A_{LL}}\;e^{j\left(\varphi_{RL,wire}-\varphi_{LL,wire}\right)}$$

where $\Delta A_{RL}$ and $\Delta A_{LL}$ are the isolated wire amplitude contributions for the RL and LL channels, respectively, obtained by subtracting the background amplitude from the wire-present amplitude at the wire’s range bin. The terms $\varphi_{RL,wire}$ and $\varphi_{LL,wire}$ are the phase angles of the RL and LL channels measured at the wire’s range bin. The amplitude ratio ($\gamma_{A}$) corrects the amplitude imbalance between the two channels, while the phase term ($\gamma_{P}$) is the phase imbalance correction. From the calibration experiment, the derived calibration factor is *γ* = 2.79 · $e^{j\left(21.20{}^{\circ}\right)}$. Because the *H*/*α* decomposition is governed by the inter-channel amplitude balance and is invariant to the absolute inter-channel phase, only the amplitude factor affects the classification. Each calibration factor is derived from an acquisition stable against external (non-hardware) noise (~1400 azimuth samples, ~3–4 s); after correction the residual imbalance has a coefficient of variation of about 5% (~0.5 dB), confirming effective channel balancing. Across two campaigns (three sessions) approximately six months apart, the channel imbalance is consistent (8.7–9.1 dB; Figure 12), in line with the low temporal variability of channel imbalance reported for airborne polarimetric SAR systems [42]. The calibrated LL signal is then obtained by multiplying the raw LL channel by *γ*, so that the LL wire contribution matches the RL reference channel.

**Figure 13:**
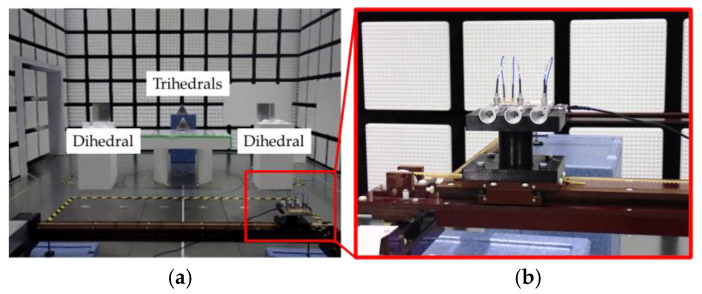
(**a**) Experimental setup in anechoic chamber with trihedral and dihedral corner reflectors, (**b**) where the inset shows the DCP antennas.

**Figure 14:**
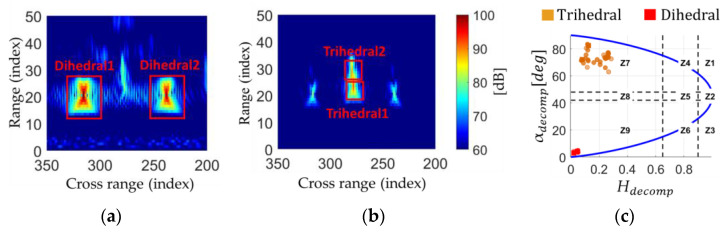
Anechoic chamber experiment results: (**a**) LL channel image, (**b**) RL channel image with decomposition region, (**c**) *H*/*α* scatter plot (blue = feasible region; dashed = zone boundaries).

To verify the calibration, trihedral and dihedral corner reflectors were measured in an anechoic chamber, Figure 13. The focused SAR images (Figure 14) confirm expected polarimetric behavior: Figure 14a shows the LL channel image where the dihedral produces a strong response, and Figure 14b shows the RL channel image with the decomposition region indicated, where the trihedral dominates. The *H*/*α* scatter plot (Figure 14c) shows clear separation between the two targets, dihedral in Zone Z9 (low *H*, low α) and trihedral in Zone Z7 (low *H*, high α), validating the wire-based calibration. Detailed decomposition results are listed in Table 3.

## 4. Experimental Results

In this section, the analysis results for the DCP-SAR dataset are presented. The dataset used in this study for the decomposition analysis includes measurements over a trihedral reflector, a dihedral reflector, an artificial object, and a natural scene (the pond and its surrounding vegetation). The first step was to apply the decomposition to the trihedral and dihedral reflectors using the drone-borne DCP SAR system to assess whether the drone-borne system can perform canonical target decomposition in a manner consistent with the tests conducted in the anechoic chamber.

### 4.1. Result of Canonical Target

**Figure 15:**
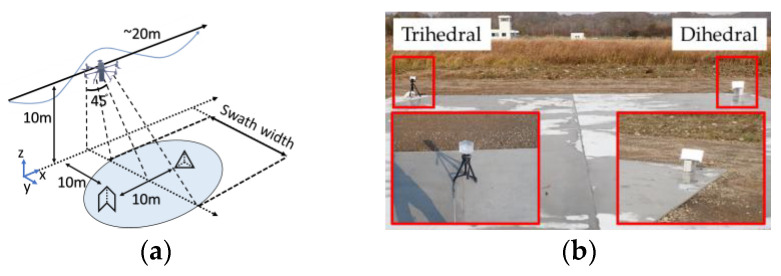
Canonical target scenario: (**a**) schematic illustration (solid = flight path; dashed = swath width), (**b**) field photograph of trihedral (left) and dihedral (right) reflectors.

Following the validation in controlled conditions, the *H*/*α* decomposition was applied to drone-borne SAR data of corner reflectors deployed in outdoor field conditions, where Figure 15a provides a schematic of the deployment geometry, and Figure 15b shows a field photograph of the trihedral (left) and dihedral (right) reflectors. This experiment aimed to evaluate decomposition performance under realistic conditions, including environmental factors such as ground clutter and vegetation.

**Figure 16:**
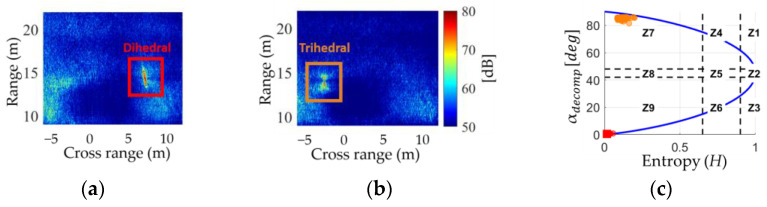
Canonical target experiment results: (**a**) LL channel image, (**b**) RL channel image, (**c**) *H*/*α* scatter plot (blue = feasible region; dashed = zone boundaries).

The dihedral corner reflector and trihedral reflector were deployed in a concrete field surrounded by grass environment. The decomposition results of the employed targets are shown in Table 4. The DCP decomposition results confirmed the correct classification of the canonical targets, as shown in Figure 16, where Figure 16a presents the LL channel image, Figure 16b the RL channel image, and Figure 16c the *H*/*α* scatter plot. The dihedral reflector, observed in the LL polarization channel (Figure 16a), was classified in Zone Z9 with very low entropy (*H* ≈ 0.021) and a near-zero alpha angle (α ≈ 0.621°), indicating a dominant even-bounce scattering mechanism.

The trihedral reflector, observed in the RL polarization channel (Figure 16b), was correctly classified in Zone Z7 with an entropy value of *H* ≈ 0.123 and a high alpha angle (α ≈ 84.873°), consistent with the expected odd-bounce scattering behavior. The scatter plot in the *H*/*α* plane (Figure 16c) clearly shows the separation between the two targets: the dihedral reflector clustered near the origin (low *H*, low *α*) in Zone Z9, while the trihedral reflector appeared in the upper-left region (low *H*, high α) in Zone Z7. Results from the drone-borne SAR experiments on the canonical targets are consistent with the anechoic-chamber measurements presented in the previous section.

**Figure 17:**
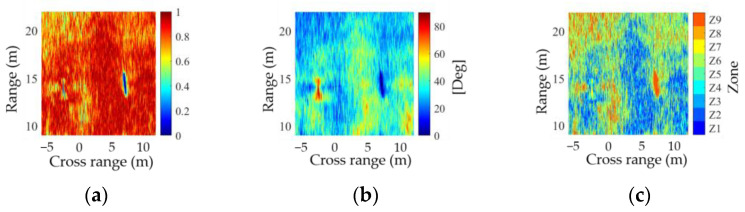
*H*/$\alpha_{DCP}$ decomposition results for trihedral and dihedral reflectors: (**a**) entropy H map, (**b**) alpha angle $\alpha_{DCP}$ map, (**c**) *H*/α zone classification map.

Figure 17 presents the *H*/*α* zone classification of the observed scene, where Figure 17a shows the entropy *H* map, Figure 17b the alpha angle $\alpha_{DCP}$ map, and Figure 17c the *H*/*α* zone classification map. The concrete surface exhibits spatially varying *H*/*α* zone distribution, with some areas dominated by Zones Z1–Z4 and others by Zones Z5–Z8; this mixed pattern likely reflects local contamination from the nearby canonical reflectors (sidelobe and smearing effects, positioning inaccuracies in the GNSS-based TDBP, or edge vegetation) rather than intrinsic heterogeneity of the concrete platform. This interpretation is supported by the artificial structure and natural scene experiments presented in the following subsections, where terrain surfaces without nearby strong point scatterers exhibit spatially consistent *H*/*α* zone classifications. The canonical reflectors are classified as reported above (trihedral: Z7; dihedral: Z9), confirming that the applied polarimetric decomposition reliably distinguishes the expected scattering mechanisms.

### 4.2. Result of Artificial Structure Target

**Figure 18:**
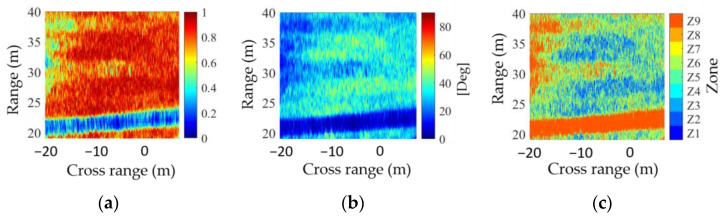
*H*/*α* decomposition results for artificial structure scene; (**a**) entropy H map, (**b**) alpha angle $\alpha_{DCP}$ map, (**c**) *H*/α zone classification map.

**Figure 19:**
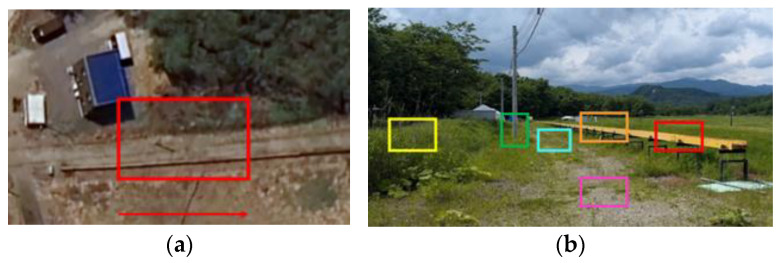
(**a**) Experiment route and observation area (Google Earth©; red arrow: drone path). (**b**) Field photograph of the artificial object with analysis regions: dense vegetation (yellow), electric pole (green), gravel road (magenta/cyan), and artificial structure (orange/red).

The *H*/*α* decomposition was further applied to characterize artificial environmental features observed in the drone-borne SAR imagery. This analysis demonstrates the practical applicability of the DCP decomposition for terrain classification and feature extraction. The *H*/*α* decomposition map results for the artificial structure are presented in Figure 18. The entropy map (Figure 18a) shows a distinct low-entropy band (*H* ≈ 0.15–0.23) at approximately Y = 20–23 m corresponding to the artificial structure, while the surrounding vegetated area (Y ≈ 24–36 m) exhibits higher entropy values (*H* ≈ 0.5–0.8). The alpha angle map (Figure 18b) confirms this distinction, with very low alpha values (α < 10°) at the structure location indicating even-bounce scattering and moderate to high values (α ≈ 30–60°) in the vegetated region. The combined *H*/*α* zone classification (Figure 18c) classifies the artificial structure predominantly in Zone Z9 (even-bounce dihedral scattering) and the vegetation in Zones Z4–Z5 (volume/mixed scattering). The corresponding scene photographs and satellite imagery are shown in Figure 19, where the drone route and observation area are indicated in Figure 19a, and the metallic artificial structure with the color analysis region highlighted is shown in Figure 19b.

**Figure 20:**
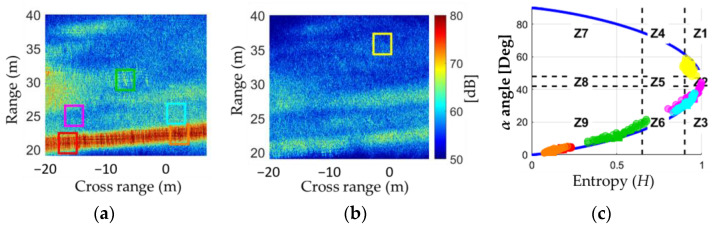
SAR focusing results for selected regions (colors corresponding to Figure 19b): (**a**) LL channel, (**b**) RL channel, (**c**) *H*/*α* scatter plot (blue = feasible region; dashed = zone boundaries).

Figure 20 shows the SAR focusing results for the selected regions, where Figure 20a and Figure 20b present the focused images in the LL and RL channels, respectively, and Figure 20c shows the corresponding H/α scatter plot. The artificial structure (red and orange markers in Figure 20c) is classified in Zone Z9 with low entropy (H ≈ 0.122–0.189) and near-zero alpha angle (α ≈ 2–4°), confirming the dominant even-bounce scattering, forming a dihedral geometry with the ground. The electric pole (green markers) also falls in Zone Z9 (H ≈ 0.509, α ≈ 13°), where the vertical metallic pole and the ground surface form a dihedral geometry that produces a dominant double-bounce response. At K-band frequencies, the pole diameter is significantly larger than the wavelength (~1.2 cm), preventing dipole-like scattering and favoring specular dihedral reflection. The gravel road with sparse vegetation (magenta and cyan markers) spans Zones Z6 and Z3 (H ≈ 0.908–0.938, α ≈ 31–36°), where the heterogeneous mixture of exposed soil and short grass produces a mixed scattering response consistent with the satellite and ground photograph imagery (Figure 19). The dense vegetation (yellow marker) exhibits high entropy and alpha angle (H ≈ 0.9281, α ≈ 52.88°) in Zone Z1, where strong depolarization arises from multiple interactions with randomly oriented elements whose size is comparable to the K-band wavelength (~1.2 cm).

### 4.3. Result of Natural Scene

**Figure 21:**
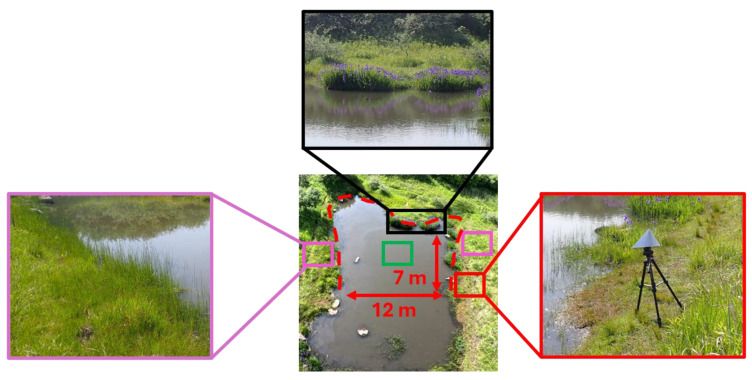
Optical image (from drone) with the four-color analysis regions (red for the trihedral reflector, magenta for vegetation, green for pond interior, black for shoreline vegetation), plus detail photographs of the trihedral CR and the surrounding vegetation.

**Figure 22:**
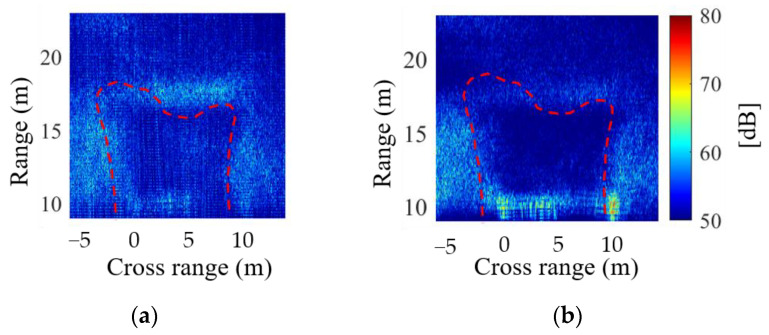
Focused SAR images in the (**a**) LL channel and (**b**) RL channel, where the red dashed line outlines the pond boundary.

In addition to artificial structures, the *H*/*α* decomposition was applied to a more challenging natural scene consisting of a pond and vegetation. Unlike the artificial structure scene, where the target is well-defined, this scene contains a heterogeneous mixture of water and vegetation that tests the decomposition’s sensitivity to subtle variations in natural surface conditions. The optical image in Figure 21, acquired from the drone, shows the pond location together with the selected color analysis regions and detailed photographs of the in-scene trihedral corner reflector and the surrounding vegetation. The pond boundary is clearly delineated in the LL polarization image (Figure 22a), while the RL channel image (Figure 22b) shows the same scene from the cross-polarization perspective. The trihedral corner reflector placed within the scene serves as an in-scene reference target with a known scattering mechanism.

**Figure 23:**
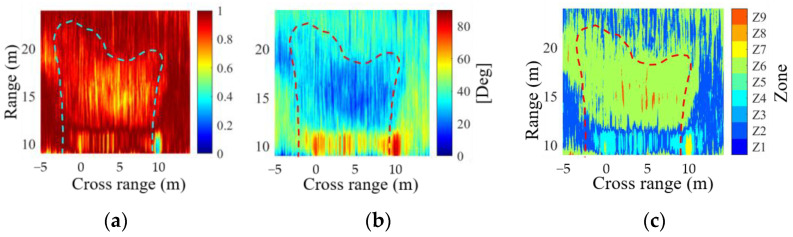
H/α decomposition for the pond scene: (**a**) entropy (H) map, (**b**) alpha angle ($\alpha_{DCP}$) map, (**c**) H/α zone classification map, where the red dashed line outlines the pond boundary.

**Figure 24:**
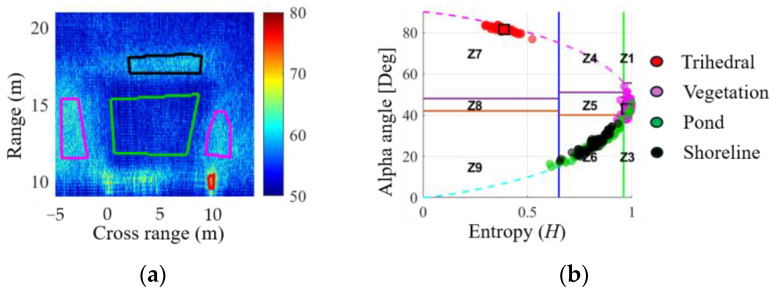
(**a**) RL channel SAR image with the four-color analysis regions, (**b**) scatter plot of the selected areas in the *H*/*α* plane (dash = feasible region; solid = zone boundaries).

The entropy map (Figure 23a), the alpha map (Figure 23b), and *H*/*α* zone classification map (Figure 23c) reveal a clear contrast between the pond and the vegetation, with the vegetated areas exhibiting predominantly high entropy, while the pond shows lower entropy and lower alpha values. This distinction is summarized in the zone classification map (Figure 23c). Four representative areas were selected (Figure 21 and Figure 24a): the trihedral reflector (red mark), the surrounding vegetation (magenta mark), the pond interior (green mark), and the shoreline vegetation (black mark). A summary of the decomposition parameters for each area is presented in Table 5, and their distribution in the *H*/*α* plane is shown in Figure 24b.

The trihedral reflector exhibits the expected low-entropy (*H* ≈ 0.39) and high-backscatter (≈67.6 dB) characteristics of an ideal odd-bounce target, consistent with the canonical-target results presented in Table 3 and Table 4. In contrast, all natural cover types exhibit high entropy. The vegetation is characterized as a high-entropy volume scatterer (*H* ≈ 0.97, Zone Z2) with balanced intensity across both polarimetric channels (≈55.7 dB LL/55.2 dB RL).

**Figure 25:**
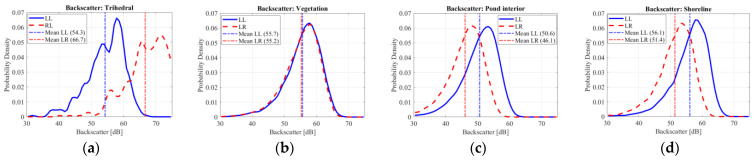
Distribution of backscatter (dB) for each analysis region: (**a**) trihedral reflector, (**b**) vegetation, (**c**) pond, (**d**) shoreline.

A specific challenge arises with the pond interior and the shoreline vegetation, as both fall into the medium-entropy Zone Z6 (*H* ≈ 0.82 and *H* ≈ 0.81, respectively), rendering polarimetric zone classification alone insufficient for their delineation. However, analyzing the channel-specific backscatter distributions (Figure 25) reveals a clear physical differentiation. The open-water pond interior exhibits weak backscatter in both channels (≈50.6 dB LL/≈46.1 dB RL). This low intensity, combined with the dark appearance in LL and RL images (Figure 22a,b), indicates a specular reflection directed away from the radar. Consistent with polarimetric observations of natural terrain, the entropy estimate here increases as the signal-to-noise ratio decreases [43]; thus, the Z6 assignment is identified as a low signal-to-noise ratio (SNR) artifact rather than a true double-bounce interaction.

Conversely, the shoreline vegetation returns significantly stronger backscatter (≈56.1 dB LL/≈51.4 dB RL), comparable to the surrounding land vegetation. Figure 21 confirms irises and reeds standing in the edge of the pond, forming a dihedral structure between the vertical stems and the water surface. The Z6 response in this area is thus a genuine even-bounce interaction, consistent with the double-bounce enhancement documented for emergent and shoreline vegetation in saturated environments [44]. This backscatter strength, particularly the robust separation observed in both LL and RL channels (Figure 25), combined with the entropy contrast against the trihedral, provides the physical basis for delineating the open water from the even-bounce shoreline.

These results show that the DCP *H*/*α* decomposition resolves the dominant scattering mechanisms of the natural scene: odd bounce for the trihedral reference (Z7), high-entropy volume scattering for the vegetation (Z2), and even bounce for the emergent shoreline vegetation (Z6). Consistent with polarimetric observations of natural terrain at this wavelength, the entropy is high for all of the natural cover and low only for the high-cross-section trihedral, and the polarimetric estimate is reliable wherever the backscatter is sufficient. The open-water pond, whose specular return is too weak for a reliable polarimetric estimate, is delineated instead by its characteristically low backscatter. These results confirm the applicability of the DCP decomposition for natural-terrain classification.

## 5. Discussion

This work demonstrates compact polarimetric *H*/*α* target decomposition on a drone-borne DCP SAR using an off-the-shelf K-band radar, showing that a single-transmit DCP configuration, which achieves a wider swath than fully polarimetric SAR for the same acquisition [24,25], can resolve odd-bounce, volume, and even-bounce scattering mechanisms for terrain classification on a drone platform. The DCP configuration distinguishes odd-bounce and even-bounce scattering with only two receive channels and a single transmit polarization more reliably than dual linear polarimetric systems with the same channel count, as DCP is inherently less sensitive to target orientation angle [1,26,27]. The experimental results confirm that the DCP *H*/*α* framework, previously validated through simulations on satellite CP data [30], extends to drone-borne platforms at K-band frequencies.

The terrain classification results demonstrate the practical value of DCP drone-borne SAR. Following wire-based calibration, artificial structures exhibit even-bounce behavior consistent with dihedral geometry, while roads produce high-entropy mixed scattering. Although *H*/*α* decomposition successfully identifies trihedral reflectors and vegetation, it encounters ambiguities in natural scenes, where both open water and shoreline vegetation fall into Zone Z6. We resolve this by incorporating channel-specific backscatter intensity: the pond’s low intensity identifies the Z6 response as a low-SNR artifact, whereas the shoreline’s higher intensity confirms a genuine even-bounce dihedral interaction. This hybrid approach provides a robust framework for terrain delineation more robust than zone classification alone. These findings align with K-band characteristics (~1.2 cm), which are well suited for surface-level classification despite limited vegetation penetration compared to L- or C-bands. Finally, as wire-based calibration only addresses receive-side imbalance, transmit-side polarization impurity remains a topic for future investigation.

These terrain classification capabilities suggest practical applicability beyond controlled experiments. In post-disaster scenarios where satellite SAR revisit delays are critical, the demonstrated capability enables rapid terrain assessment, where distinguishing structural damage, flood extent, and debris from intact terrain relies on the same scattering mechanism classification demonstrated in this study. Furthermore, by conducting a wide-swath drone swarm operation, it becomes possible to rapidly monitor a large area during disasters.

## 6. Conclusions

This work applies compact polarimetric *H*/*α* decomposition to DCP SAR data acquired from a drone-borne platform using an off-the-shelf K-band radar. Three contributions are established: (1) coherent image formation through GNSS-guided TDBP with PGA, yielding an 11.98 dB peak amplitude improvement and a 2.32 cm half-power beam width; (2) a single-target wire calibration correcting an 8.91 dB inter-channel gain difference; and (3) H/α discrimination of canonical reflectors and multiple terrain types, including artificial structures, gravel roads, and vegetation, as well as the delineation of a water surface, whose weak radar return is itself a distinguishing low-backscatter signature.

## Figures and Tables

**Table 1:** Specifications of Hardware.

Name	Value
Frequency SAR	24.15 GHz
Polarization	LL/RL
Sampling frequency	1.8 MHz
Beamwidth	45° (cross-range)
Bandwidth	180 MHz

**Table 2:** Alpha angle correspondence between FP and DCP modes for canonical targets.

Scattering Mechanism	Target Example	$\alpha_{FP}$	${\overset{‾}{\alpha }}_{DCP}$
Odd-bounce (surface)	Trihedral, Sphere, Plate	0°	90°
Wire	Wire, Thin cylinder	45°	45°
Even-bounce (double)	Dihedral	90°	0°

**Table 3:** *H*/*α* decomposition results for anechoic chamber reflectors.

Reflector	Entropy (*H*)	${\overset{‾}{\alpha }}_{DCP}$ (deg)	Zone	Classification
Dihedral1	~0.021	~2.962°	Z9	Low entropy even bounce
Dihedral2	~0.045	~4.328°	Z9	Low entropy even bounce
Trihedral1	~0.123	~82.451°	Z7	Low entropy odd bounce
Trihedral2	~0.080	~71.989°	Z7	Low entropy odd bounce

**Table 4:** H/α decomposition results for outdoor canonical targets.

Reflector	Entropy (*H*)	${\overset{‾}{\alpha }}_{DCP}$ (deg)	Zone	Classification
Dihedral (Red)	~0.021	~0.621°	Z9	Low entropy even bounce
Trihedral (Orange)	~0.123	~84.873°	Z7	Low entropy odd bounce

**Table 5:** Mean values and standard deviations of DCP H/α decomposition parameters and dominant scattering mechanisms for observed pond scene features.

Region	Backscatter LL (dB)	Backscatter RL (dB)	Entropy *H*	Mean α (°)	Zone	Dominant Mechanism
Trihedral reflector	54.3 ± 6.2	66.7 ± 6.1	0.39 ± 0.05	81.4 ± 1.3	Z7	Odd bounce (reference)
Vegetation	55.7 ± 5.6	55.2 ± 5.7	0.97 ± 0.02	42.9 ± 4.8	Z2	High-entropy volume
Pond (water)	50.6 ± 5.7	46.1 ± 5.7	0.82 ± 0.09	26.4 ± 6.3	Z6	Open water (low backscatter)
Shoreline (emergent veg.)	56.1 ± 5.6	51.4 ± 5.5	0.81 ± 0.06	26.0 ± 3.7	Z6	Even bounce (medium entropy)

## Data Availability

The raw SAR datasets and processing scripts used in this study are available from the corresponding author upon reasonable request.

## Abbreviations

The following acronyms are used in this manuscript:

SAR	Synthetic Aperture Radar
GNSS	Global Navigation Satellite System
DCP	Dual Circular Polarization
FMCW	Frequency-Modulated Continuous Wave
LHCP	Left-Hand Circular Polarization
RHCP	Right-Hand Circular Polarization
TDBP	Time-Domain Back-Projection
PGA	Phase Gradient Autofocus
LL	LHCP-receive LHCP-transmit
RL	RHCP-receive LHCP-transmit
PRF	Pulse Repetition Frequency
FP	Fully Polarimetric
CP	Compact Polarimetric
DLP	Dual Linear Polarimetric
SNR	Signal-to-Noise Ratio
